# A risk model for prediction of diagnosis of cancer after ischemic stroke

**DOI:** 10.1038/s41598-022-26790-y

**Published:** 2023-01-03

**Authors:** Katharina Seystahl, Dorothee Gramatzki, Miriam Wanner, Sung Ju Weber, Alessia Hug, Andreas R. Luft, Sabine Rohrmann, Susanne Wegener, Michael Weller

**Affiliations:** 1 Department of Neurology, University Hospital and University of Zurich, CH-8091 Zurich, Switzerland; 2 Cancer Registry of the Cantons of Zurich, Zug, Schaffhausen and Schwyz, University Hospital and University of Zurich, Zurich, Switzerland; 3 Cereneo Center for Neurology and Rehabilitation, Vitznau, Switzerland

**Keywords:** Cancer screening, Stroke

## Abstract

It remains controversial which characteristics may predict occult cancer in stroke patients. Characteristics of patients with ischemic stroke registered in the Zurich Swiss Stroke Registry (2014 to 2016) were tested for associations with cancer diagnosis after stroke with consideration of death as competing risk for cancer diagnosis. Among 1157 patients, 34 (3%) and 55 patients (5%) were diagnosed with cancer within 1 and 3 years after stroke. Levels of white blood cells (WBC) > 9,600/µl (subdistribution hazard ratio (SHR) 3.68, *p* = 0.014), platelets > 400,000/µl (SHR 7.71, *p* = 0.001), and d-dimers ≥ 3 mg/l (SHR 3.67, *p* = 0.007) were independently associated with cancer diagnosis within 1 year after stroke. Occurrence of ischemic lesions in ≥ 2 vascular territories not attributed to cardioembolic etiology was associated with cancer diagnosed within 1 year after stroke in univariable analysis (SHR 3.69, *p* = 0.001). The area under the curve of a score from these parameters (score sum 0–4) was 0.73. A score of ≥ 2 had a sensitivity of 43% and specificity of 92% for prediction of cancer diagnosis within 1 year after stroke. We suggest further validation of a score of WBC, platelets, d-dimers and multiple ischemic lesions without cardioembolic stroke etiology for prediction of cancer diagnosis after stroke.

## Introduction

Cerebrovascular disease is common among patients with cancer with an increased risk of stroke accumulating around the time of cancer diagnosis^1^. A historical autopsy study of 3,426 patients with systemic cancer revealed cerebrovascular disease, i.e. hemorrhages or ischemic infarction, in 500 patients (14.6%); 245 of 500 strokes (49%) were clinically silent^2^. Beyond cancer as a risk factor for stroke, several studies have explored whether stroke may precede the diagnosis of cancer, with the aim to identify patients with occult cancer. A review of 51 articles evaluating patients with ischemic stroke and occult cancer calculated a cumulative incidence of cancer stroke of 1.4% (95% confidence interval (CI), 0.6–2.5) within 1 year after ischemic stroke. The cumulative incidence rate was higher in studies with dedicated cancer screening activities (3.9%, 95% CI 1.6–7.1) suggesting that occult cancer may be underdiagnosed in the context of acute ischemic stroke^3^. In an observational study with 37,000 patients followed for up to 10 years in general practices in Germany, risk of cancer diagnosis after stroke was higher in patients with a history of stroke compared to those without^4^. Similar to this finding, a post-hoc analysis of a prospective study observed a higher age-adjusted annual rate of cancer at 1 or 2 years after stroke in patients with ischemic stroke than in the general population^5^.

Despite previous studies acknowledging the clinical importance of identifying patients with occult cancer, it remains controversial whether there are specific risk factors in stroke patients that would justify screening for cancer. Several risk factors associated with occult cancer have been suggested, among them older age, smoking, involvement of multiple vascular territories, elevated levels of C-reactive protein (CRP), d-dimers, and lower levels of hemoglobin^3,6,7^.

Most studies represent single institution retrospective analyses focusing on the early post-stroke phase and methodologically do not consider death as competing risk for cancer diagnosis after stroke. The latter is especially important since higher in-hospital mortality and inferior post-stroke survival is associated with cancer in patients with acute ischemic stroke^5,8,9^.

The present study represents an exploratory analysis of potential predictive factors associated with risk of cancer diagnosis after acute ischemic stroke without and with consideration of death as a competing risk for a diagnosis of cancer. To consider uncertainties regarding a potential temporal relationship between the diagnosis of stroke and occult cancer, we performed different analyses for patients diagnosed with cancer within 1 year or 3 years after stroke.

## Patients and methods

### Patients

Patients with acute ischemic stroke diagnosed from 2014 to 2016 were identified by the Swiss Stroke Registry of Zurich with institutional review board approval (KEK-ZH 2018-01917). Patients’ medical records were retrospectively analyzed for disease characteristics, laboratory parameters and outcome. Incidence and characteristics of cancer and date of follow-up was retrieved as available in the medical records for all patients and matched with the data of the Cancer Registry of the Cantons of Zurich, Zug, Schaffhausen and Schwyz in Switzerland, where applicable. Subsets of patients with ischemic stroke have been previously described^8,10^.

### Variables

Cancer after stroke was recorded if any neoplastic disease was diagnosed after acute ischemic stroke excluding benign tumors such as adenomas, basal cell carcinoma, schwannomas and meningiomas. Patient demographics and clinical data including stroke severity via the National Institutes of Health Stroke Scale (NIHSS) were derived from the clinical chart. As medical history, conditions known prior to stroke were considered. Stroke etiology was assessed by the TOAST classification^11^ based on the medical records. Diagnostic work-up for stroke etiology commonly included neuroimaging with vessel imaging by computed tomography (CT) or magnetic resonance (MR) angiography, cardiac studies with 12-channel ECG, telemetry and/or Holter ECG monitoring for at least 48 h, and transthoracic or transesophageal echocardiography, as well as duplex ultrasound of the neck and intracranial arteries. For the incidence of large vessel occlusions the first vessel imaging by CT or magnetic resonance MR angiography was used. For evaluation of ischemic lesions in different vascular territories, MR of the brain was used if available and CT if no MRI was done. Criteria of stroke-associated infections, i.e. pneumonia, urinary tract infection, and other infections diagnosed within 7 days after stroke onset were used as described^10^. For laboratory parameters, the first value available after admission was derived from the medical records and only included if obtained within 24 h after admission for stroke except for lactate dehydrogenase (LDH) where later sample acquisition was accepted. For d-dimers and fibrinogen, values obtained after intravenous thrombolysis were excluded. The following local standard upper level of normal (ULN) were used: CRP 5 mg/dl, white blood cell count (WBC) 9,600/μl, platelet count 400,000/μl, LDH 480 U/l, d-dimers 0.5 mg/l, and fibrinogen 4.0 g/l. For hemoglobin, 117 g/l and 134 g/l were used as lower level of normal (LLN) in women and men, respectively.

### Statistical analysis

Chi-square test was used for comparison of categorical and Mann–Whitney U tests for ordinal and continuous variables. Laboratory parameters were used as continuous and as categorical variables using the LLN and ULN, respectively, as cut-offs.

Cox proportional hazards regression analysis was performed to calculate the risk of cancer diagnosis after stroke using the time of diagnosis of stroke to the cancer diagnosis of cancer after stroke. Patients who were not diagnosed with cancer after stroke were censored at the date of last follow-up or death. For competing risk analyses with death as competing risk for cancer diagnosis, proportional subdistribution hazard ratios (SHR) were estimated by the Fine and Gray model^12^. Multivariable models were calculated in a subgroup of patients with all tested co-variables available. Receiver operating characteristic (ROC) curves and area under the curve (AUC) were calculated to rate score validity^13^ with an AUC of 1 for perfect discrimination capacity and an AUC of 0.5 corresponding to chance discrimination. A *p* value of < 0.05 was defined as significant. Statistical analyses were performed using SPSS Statistics, Version 26, Stata/SE, Version 16, and Graphpad Prism, Version 8.0.

### Ethical approval

This study was performed in line with the principles of the Declaration of Helsinki. Approval for the study was received by the local institutional review board Cantonal ethics committee Zurich (KEK-ZH 2018-01917).

### Consent to participate

Patient data were used with consent available or not needed according to the requirements of the institutional review board Cantonal ethics committee Zurich approval (KEK-ZH 2018-01917).

## Results

### Patient characteristics

**Figure 1:**
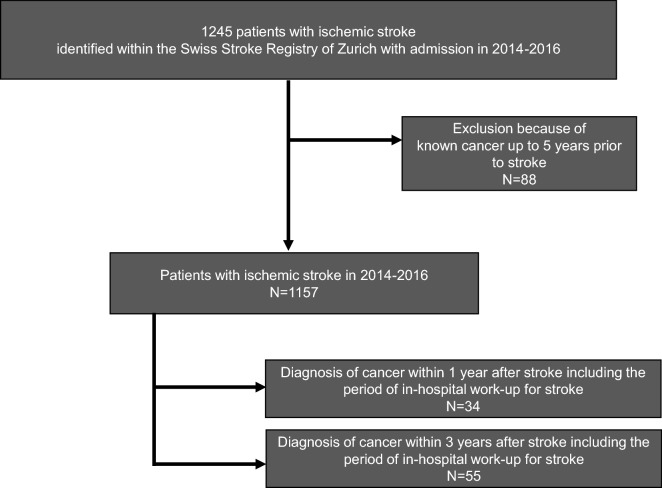
Consort chart. Process of patient identification for the study cohort.

1245 patients with ischemic stroke were identified from the Zurich Swiss Stroke Registry between 2014 and 2016 (Fig. 1). To identify specific characteristics associated with risk of diagnosis of cancer after stroke with patients without cancer diagnosis, we excluded patients with cancer diagnosed up to 5 years prior to stroke (n = 88). Out of 1157 remaining patients, 34 patients (2.9%) were diagnosed cancer within 1 year after stroke including the period of in-hospital work-up of stroke and 55 patients (4.8%) within 3 years after stroke (Table 1). The most frequent cancer types diagnosed after stroke were lung cancer, lymphoma and other hematologic diseases, and prostate cancer. Lymph node and/or distant metastases were documented in about one third of patients. Clinical pathways leading to the diagnosis of cancer in patients during hospitalization for stroke (n = 20) were variable, in three patients (15%), cancer represented an incidental finding, in 12 patients (60%), targeted tests were performed due to clinical suspicion of cancer triggered by clinical findings and/or laboratory findings, and in 5 patients (25%), CT of chest and abdomen or whole body fluorodeoxyglucose positron emission tomography were performed for general suspicion or differential diagnosis of cancer (Table 1).

**Table 1:** Characteristics of patients diagnosed with cancer after ischemic stroke.

	Cancer diagnosed within 1 year after stroke: 34 patients	Cancer diagnosed within 3 years after stroke: 55 patients
Median time from stroke to cancer diagnosis: months, 95% CI	0.5 (0.2–4.8)	7.3 (1.5–12.9)
Diagnosis of cancer during hospitalization for ischemic stroke: n (%)	20 (58.8%)	20 (36.4%)
**Diagnostic pathways leading cancer diagnosis during hospitalization for stroke:**		
(1) Cancer as incidental finding without prior suspicion of cancer: n (%)	3/20 (15%)	3/20 (15%)
(2) Targeted diagnostics done due to symptoms or laboratory findings prompting suspicion for cancer: n (%)	12/20 (60%)	12/20 (60%)
(3) CT scan of chest and abdomen or whole body fluorodeoxyglucose positron emission tomography for differential diagnosis of cancer	5/20 (25%)	5/20 (25%)
**Type of cancer (first diagnosis after stroke) n (%)**		
Lung cancer	6 (17.6%)	8 (14.5%)
Lymphoma and hematologic diseases	6 (17.6%)	8 (14.5%)
Prostate cancer	3 (8.8%)	5 (9.1%)
Head and neck cancer	2 (5.9%)	4 (7.3%)
Cancer of unknown origin	3 (8.8%)	3 (5.5%)
Breast cancer	2 (5.9%)	3 (5.5%)
Pancreatic cancer	2 (5.9%)	3 (5.5%)
Colorectal cancer	2 (5.9%)	3 (5.5%)
Skin cancer	2 (5.9%)	2 (3.6%)
Urothelial cancer	1 (2.9%)	6 (10.9%)
Kidney cancer	1 (2.9%)	2 (3.6%)
Sarcoma	1 (2.9%)	2 (3.6%)
Gastric cancer	1 (2.9%)	1 (1.8%)
Central nervous system	1 (2.9%)	1 (1.8%)
Biliary tract cancer	1 (2.9%)	1 (1.8%)
Ovarian cancer	–	1 (1.8%)
**Second cancer after stroke**	3 (8.8%)	6 (10.9%)
Ovarian cancer	1 (2.9%)	1 (1.8%)
Lymphoma and hematologic diseases	1 (2.9%)	2 (3.6%)
Urothelial cancer	1 (2.9%)	1 (1.8%)
Lung cancer		1 (1.8%)
Prostate cancer		1 (1.8%)
**Lymph node metastasis: n (%)**		
Yes	13 (38.2%)	17 (30.9%)
No	8 (23.5%)	18 (32.7%)
No data	13 (38.2%)	20 (36.4%)
**Distant metastasis: n (%)**		
Yes	13 (38.2%)	16 (29.1%)
No	11 (32.4%)	24 (42.6%)
No data	10 (29.4%)	15 (27.3%)
**Any tumor-related therapeutic intervention including surgery or systemic therapy or radiotherapy after stroke: n (%)**		
Yes	15 (44.1%)	27 (49.1%)
Systemic therapy	9 (26.5%)	13 (23.6%)
Radiotherapy	4 (11.8%)	4 (7.3%)
No	8 (23.5%)	10 (18.2%)
No data	11 (32.4%)	18 (32.7%)

Comparing patients diagnosed with cancer within 1 year and 3 years after stroke with the respective control cohort without cancer, characteristics were similar except of a higher rate of previous venous thromboembolism in patients diagnosed with cancer within 1 year after stroke (*p* = 0.011) and a lower rate of atrial fibrillation in patients with cancer diagnosed within 3 years after stroke (*p* = 0.009) (Table 2). Stroke etiology was different in patients without versus with cancer diagnosed within 1 year (*p* = 0.001) and within 3 years after stroke (*p* = 0.012). For patients diagnosed with cancer within 1 year after stroke, in 7 patients (20.6%) stroke etiology was “other determined etiology”, i.e. TOAST IV, among them 6 with cancer as respective etiology, all of them diagnosed during in-hospital work-up of stroke. Cardioembolic stroke (TOAST II) was less frequent in patients diagnosed with cancer within 1 and 3 years after stroke (*p* = 0.013 and *p* = 0.004). Occurrence of ischemic lesions in ≥ 2 vascular territories not attributed to a cardioembolic etiology was more frequent in patients diagnosed with cancer within 1 year after stroke (*p* < 0.001).

**Table 2:** Patient characteristics.

	No cancer within 1 year after stroke: 1123 patients	Cancer within 1 year after stroke: 34 patients	*p* value	No cancer within 3 years after stroke: 1102 patients	Cancer within 3 years after stroke: 55 patients	*p* value
**Sex: n (%)**			0.11			0.21
Male	638 (56.8%)	24 (70.6%)		626 (56.8%)	36 (65.5%)	
Female	485 (43.2%)	10 (29.4%)		476 (43.2%)	19 (34.5%)	
**Age: Median (Min–max)**	73 (19–100)	75 (40–95)	0.47	73 (19–100)	74 (40–95)	0.34
**NIHSS (admission)**			0.30			0.27
Available data: n %	1096 (97.5%)	33 (97.1%)		1075 (97.5%)	54 (98.1%)	
Median (Min–max)	5 (0–30)	4 (0–20)		5 (0–30)	4 (0–20)	
**Medical history: n (%)**						
Venous thromboembolism prior to stroke	55 (4.9%)	5 (14.7%)	*0.011	55 (5.0%)	5 (9.1%)	0.18
Arterial hypertension	763 (67.9%)	22 (64.7%)	0.69	746 (67.7%)	39 (70.9%)	0.62
Diabetes mellitus	175 (15.6%)	4 (11.8%)	0.54	173 (15.7%)	6 (10.9%)	0.34
Hyperlipidemia	567 (50.5%)	15 (44.1%)	0.46	558 (50.6%)	24 (43.6%)	0.31
Active or previous smoking			0.62			0.63
- Yes	435 (38.7%)	15 (44.1%)		426 (38.7%)	24 (43.6%)	
- No	624 (55.6%)	18 (52.9%)		612 (55.5%)	30 (54.5%)	
- Data available	1059 (94.3%)	33 (97.1%)		1038 (94.2%)	54 (98.2%)	
Atrial fibrillation (including diagnoses obtained during work-up of stroke)	322 (28.7%)	5 (14.7%)	0.08	320 (29.0%)	7 (12.7%)	*0.009
Ischemic stroke	152 (13.5%)	6 (17.6%)	0.49	148 (13.4%)	10 (18.2%)	0.32
**Acute therapy for stroke**						
Intravenous thrombolysis	372 (33.1%)	7 (20.6%)	0.13	368 (33.4%)	11 (20.0%)	*0.04
Intraarterial therapeutic intervention	152 (13.5%)	5 (14.7%)	0.84	151 (13.7%)	6 (10.9%)	0.56
**TOAST classification**			*0.001^1^			*0.012^1^
(1) Large artery atherosclerosis	178 (15.9%)	3 (8.8%)	0.27^2^	171 (15.5%)	10 (18.2%)	0.60^2^
(2) Cardiac embolism	395 (35.2%)	5 (14.7%)	*0.013^2^	391 (35.5%)	9 (16.4%)	*0.004^2^
(3) Small vessel disease	116 (10.2%)	6 (17.6%)	0.17^2^	112 (10.2%)	10 (18.2%)	0.06^2^
(4) Other determined etiology including cancer	66 (5.9%)	7 (20.6%)	n/a^3^	66 (6.0%)	7 (12.7%)	n/a^3^
(5) Unknown etiology or multiple possible etiologies	368 (32.8%)	13 (38.2%)	0.50^2^	362 (32.8%)	19 (34.5%)	0.79^2^
**Ischemic lesions in ≥ 2 vascular territories**	174 (15.5%)	9 (26.5%)	0.08	173 (15.7%)	10 (18.2%)	0.6
**Ischemic lesions in ≥ 2 vascular territories not attributed to cardioembolic stroke etiology (TOAST 2)**	97 (8.6%)	9 (26.5%)	* < 0.001	97 (8.8%)	9 (16.4%)	0.06
**Large vessel occlusion**			0.27			0.10
Yes	407 (36.2%)	9 (26.5%)		402 (36.5%)	40 (72.7%)	
No	705 (62.8%)	24 (70.6%)		689 (62.5%)	54 (98.2%)	
Data available	1112 (99.0%)	33 (97.1%)		1091 (99.0%)	14 (25.5%)	
**Stroke-associated infections**	183 (16.3%)	7 (20.6%)	0.51	181 (16.4%)	9 (16.4%)	0.99

^1^Group comparison, ^2^Single comparisons, ^3^Analysis omitted.

**Figure 2:**
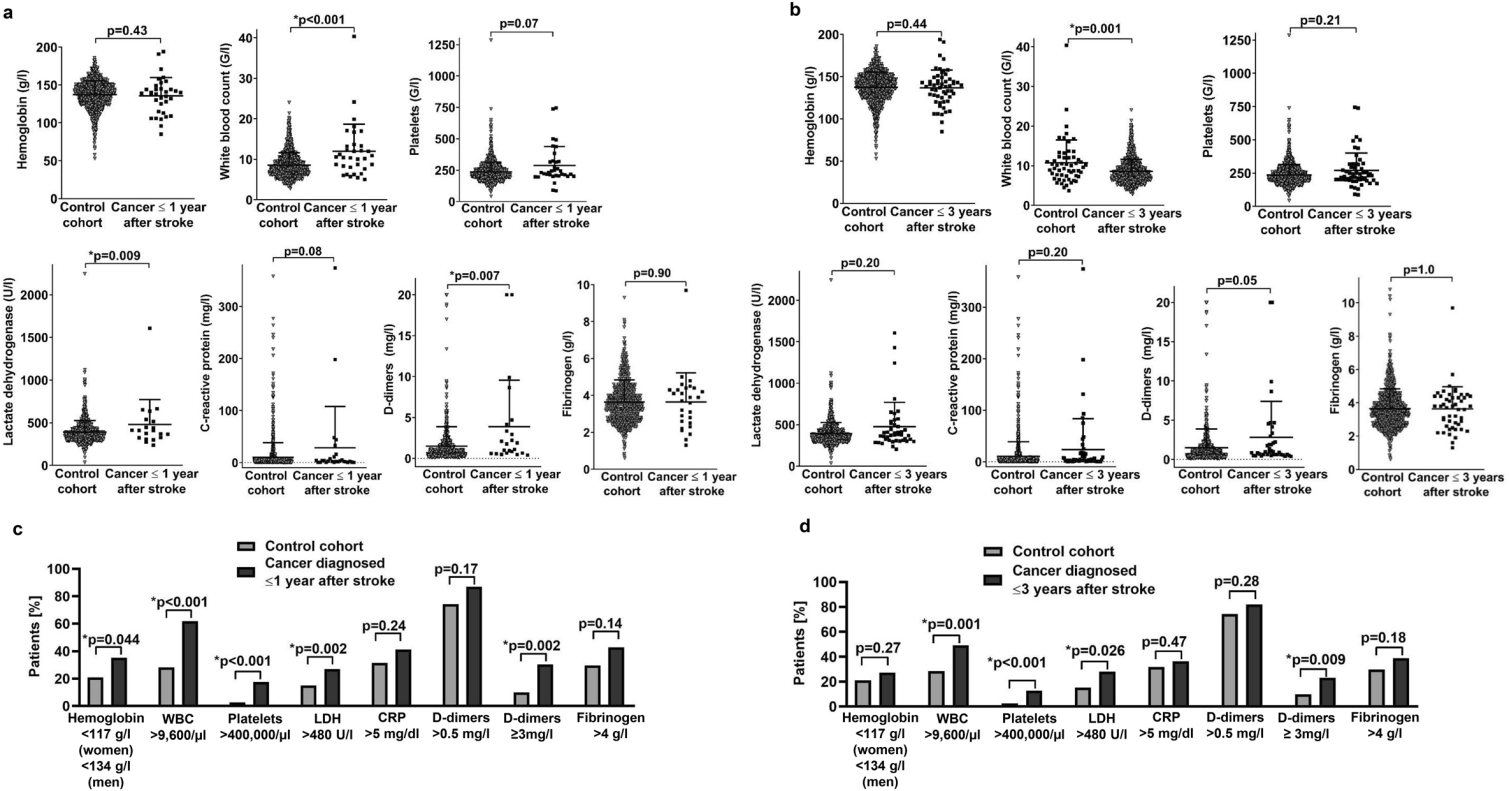
Laboratory parameters of patients with ischemic stroke without and with cancer diagnosed after stroke. (**a**–**d**). Laboratory parameters with the first value available after admission for stroke were analyzed as continuous parameters by Mann–Whitney U test (**a**,**b**) and as categorical variables using the local standard upper or lower level of normal as cut-offs by Chi-Square test (**c**,**d**) comparing patients without and with cancer diagnosed within 1 year (**a**,**c**) and 3 years after stroke (**b**,**d**). Data were shown as scatter plots including mean and SD (**a**,**b**) and as bars with percentages (**b**,**d**) for patients with data on the indicated laboratory parameter available, i.e. out of 1157 patients for 1157 patients for hemoglobin, white blood count and platelets, for 909 for lactate dehydrogenase, for 1151 for C-reactive protein, for 794 patients for d-dimers, and for 944 patients for fibrinogen.

We next evaluated whether alterations in laboratory parameters including hemoglobin, WBC, platelets, LDH, CRP, d-dimers, and fibrinogen were associated with cancer diagnosed after stroke. We observed that higher levels of WBC (*p* < 0.001), LDH (*p* = 0.009), and d-dimers (*p* = 0.007) were associated with cancer diagnosis within 1 year after stroke as well as higher levels of WBC (*p* = 0.001) with cancer diagnosis within 3 years after stroke (Fig. 2a,b). Using the ULN or LLN as cut-off, WBC > 9,600/µl (*p* < 0.001 and *p* = 0.001), platelets > 400,000/µl (*p* < 0.001 and *p* < 0.001), and LDH > 480 U/l (*p* = 0.002 and *p* = 0.026) were associated with cancer diagnosed within 1 year and 3 years after stroke. Hemoglobin levels lower than the LLN (117 g/l for women, 134 g/l for men) were associated with cancer diagnosis within 1 year after stroke (*p* = 0.044) (Fig. 2c,d). Based on previous studies suggesting higher cut-off values of d-dimers, among them 0.82 mg/l^14^, 1.2 mg/l^15^, and 3 mg/l^6^ to be associated with occult cancer, we added analyses with these cut-offs and observed that levels of d-dimers ≥ 3 mg/l (*p* = 0.002 and *p* = 0.009, Fig. 2c,d) were associated with cancer diagnosed within 1 year and 3 years after stroke in our cohort while levels of d-dimers higher than 0.82 mg/l or higher than 1.2 mg/l as suggested before did not show differences between groups (data not shown).

Alterations in hematologic parameters and inflammatory biomarkers may be unspecific and may be overrepresented in patients with stroke-associated infections. We therefore evaluated the frequency of stroke-associated infections in patients with and without cancer diagnosed within 1 and 3 years after stroke which was not significantly different (Table 2). Since lymphomas and other hematologic diseases were among the most frequent entities and alterations of hematologic parameters may be relatively more frequent for them than for other malignancies, we performed separate analysis with exclusion of these patients (n = 6). In this subcohort, we also observed that higher levels of WBC (*p* = 0.009), LDH (*p* = 0.016), and d-dimers (*p* = 0.002) were associated with cancer diagnosis within 1 year after stroke as well as higher levels of WBC (*p* = 0.019) with cancer diagnosis within 3 years after stroke (Figure S1a,b). Higher levels of CRP were associated with cancer diagnosed within 1 and 3 years after stroke (*p* = 0.02 and *p* = 0.042) as well as higher d-dimers were associated with cancer diagnosed within 3 years after stroke (*p* = 0.019) (Figure S1a, b). Using the ULN or LLN as cut-off, results in this subcohort with exclusion of patients with lymphomas and hematologic diseases were comparable to the analyses in the entire cohort except for levels of platelets which were not significantly different (Figure S1c, d).

We hypothesize that a pathophysiologic relationship of the analyzed parameters and cancer may be more likely if the time interval between stroke and diagnosis of cancer is shorter since cancer is then more likely to be occult at the time of stroke. Therefore, we performed a sensitivity analysis comparing characteristics of patients diagnosed with cancer within 1 year after stroke with those patients diagnosed with cancer more than 1 year after stroke (but within 3 years after stroke) (Table S2). Higher levels of WBC (*p* = 0.017) as well as occurrence of ischemic lesions in ≥ 2 vascular territories were more frequent in patients with earlier cancer diagnosis, i.e. within 1 year after stroke while the other parameters were similar between groups.

### Association of candidate parameters with risk for cancer diagnosis after stroke

Next, we analyzed the association of parameters with risk of cancer diagnosis after stroke without and with consideration of death as a competing risk for cancer diagnosis. Out of 1157 patients, 296 patients (25.6%) died during follow-up with a median follow-up of 2 years in surviving patients.

In univariable analysis, history of venous thromboembolism (Hazard ratio (HR) 3.16, *p* = 0.018 and SHR 3.14, *p *= 0.018), levels of d-dimers ≥ 3 mg/l (HR 4.62, *p* = 0.001 and SHR 3.88, *p* = 0.003), elevated levels of WBC (HR 4.17, *p* < 0.001 and SHR 3.98, *p* < 0.001), elevated levels of platelets (HR 7.92, *p* < 0.001 and SHR 7.46, *p* < 0.001), elevated levels of LDH (HR 3.50, *p* = 0.002 and SHR 3.14, *p* = 0.004), and low levels of hemoglobin (HR 2.30, *p* = 0.02 and SHR 2.08, *p* = 0.041) were associated with risk of cancer diagnosis within one year without and with consideration of death as competing risk for cancer diagnosis (Table S1). Patients classified to a cardioembolic etiology of stroke were less likely to be diagnosed with cancer within 1 year after stroke in competing risk analysis (HR 0.34, *p* = 0.025 and SHR 0.32, *p* = 0.018). The occurrence of ischemic lesions in ≥ 2 vascular territories itself was not associated with risk of cancer diagnosis within 1 year after stroke, however, if stroke was not attributed to a cardioembolic etiology (TOAST 2), this was significant (HR 3.92, *p* < 0.001 and SHR 3.69, *p* = 0.001).

For risk of cancer diagnosis within 3 years after stroke, there was an association with levels of d-dimers ≥ 3 mg/l (HR 3.47, *p* = 0.001 and SHR 2.67, *p* = 0.011), elevated levels of WBC (HR 2.48, *p* = 0.001 and SHR 2.38, p = 0.001), elevated levels of platelets (HR 5.68, *p* < 0.001 and SHR 5.13, *p* < 0.001), and elevated levels of LDH (HR 2.27, *p* = 0.015 and SHR 1.98, *p* = 0.049). Diagnosis of cancer within 3 years after stroke was less likely in patients with atrial fibrillation (HR 0.44, *p* = 0.042 and SHR 0.36, *p* = 0.012) and in patients with a cardioembolic etiology of stroke (HR 0.39, *p* = 0.01; SHR 0.35, *p* = 0.004) (Table S1. The occurrence of ischemic lesions in ≥ 2 vacular territories not attributed to a concurrent cardioembolic etiology was associated with risk of cancer diagnosis within 3 years after stroke (HR 2.17, *p* = 0.034), while this association was not significant in competing risk analyses (Table S1).

Stroke-associated infections as potential confounder for alterations in peripheral blood count and inflammatory biomarkers were not associated with differences in risk of cancer diagnosed within 1 and 3 years after stroke in patients with and without consideration of death as competing risk for cancer diagnosis (Table S1).

To assess which parameters were independently associated with risk of cancer diagnosis after stroke without and with consideration of death as a competing risk, we included parameters with a *p*-value < 0.05 for an association with risk of cancer diagnosed within 1 year after stroke in univariable analysis (Table S1) as well as age and sex as possible confounders in multivariable models of Cox proportional hazard and competing risk analyses (Table 3). To avoid circularity bias and semantic overlap of parameters in this model, we included “ischemic lesions in ≥ 2 vascular territories not attributed to cardioembolic stroke” as parameter while atrial fibrillation and stroke etiologies by TOAST were not included as separate items. Regarding the cut-off of levels of d-dimers, we used the previosuly suggested cut-off of 3 mg/l^6^ which was associated with better discrimination of groups than other cut-offs. Risk of cancer diagnosis within 1 year was independently associated with elevated levels of WBC (HR 3.96, *p* = 0.007; SHR 3.68, *p* = 0.014), platelets (HR 7.86, *p* = 0.001; SHR 7.711, *p* = 0.001), and levels of d-dimers ≥ 3 mg/l (HR 4.40, *p* = 0.008 and SHR 3.67, *p* = 0.007).

**Table 3:** Risk of cancer diagnosis after stroke without and with consideration of death as competing risk in multivariable analyses.

	Risk of cancer diagnosis within 1 year after stroke Data for 651 patients with all covariables available				Risk of cancer diagnosis within 3 years after stroke Data for 651 patients with all covariables available			
	Hazard ratio (95% CI)	*p* value	Subdistribution hazard ratio (95% CI)	*p* value	Hazard ratio (95% CI)	*p* value	Subdistribution hazard ratio (95% CI)	*p* value
**Sex** Male versus female (ref)	1.94 (0.72–5.19)	0.19	2.0 (0.67–5.99)	0.22	1.24 (0.60–2.62)	0.56	1.28 (0.56–2.93)	0.56
**Age**	1.04 (1.0–1.09)	0.07	1.03 (0.99–1.08)	0.11	1.04 (1.00–1.07)	*0.026	1.03 (1.0–1.05)	0.07
**White blood cell count** > 9,600/µl versus ≤ 9,600/µl (ref)	3.96 (1.46–10.77)	*0.007	3.68 (1.30–10.40)	*0.014	2.56 (1.21–5.41)	*0.044	2.45 (1.18–5.07)	*0.016
**Platelet count** > 400,000/µl versus ≤ 400,000/µl (ref)	7.86 (2.28–27.10)	*0.001	7.71 (2.44–24.41)	*0.001	4.11 (1.36–12.39)	*0.012	3.95 (1.39–11.27)	*0.01
**Hemoglobin** < 117 g/l (for women) and < 134 g/l (for men) versus > 117 g/l (for women) and for > 134 g/l (men) (ref)	1.57 (0.59–4.12)	0.36	1.47 (0.56–3.85)	0.43	1.49 (0.67–3.34)	0.33	1.29 (0.59–2.82)	0.53
**Lactate dehydrogenase** > 480 U/l versus ≤ 480 U/l (ref)	1.42 (0.52–3.89)	0.39	1.63 (0.64–4.19)	0.31	1.18 (0.52–2.71)	0.69	1.28 (0.54–3.03)	0.57
**D-dimers** ≥ 3 mg/l versus < 3 mg/l (ref)	4.40 (1.48–13.13)	*0.008	3.67 (1.42–9.50)	*0.007	2.54 (1.05–6.17)	*0.039	2.13 (0.97–4.68)	0.06
**Venous thromboembolism prior to stroke** Yes versus no (ref)	2.46 (0.78–8.36)	0.12	2.78 (0.99–7.82)	0.05	1.59 (0.53–4.75)	0.40	1.76 (0.61–5.05)	0.30
**Ischemic lesions in ≥ 2 vascular territories not attributed to cardioembolic etiology (TOAST 2)** Yes versus no (ref)	2.70 (0.81–8.98)	0.10	2.51 (0.79–7.98)	0.12	1.63 (0.55–4.86)	0.38	1.51 (0.51–4.48)	0.46

Risk of diagnosis of cancer within 3 years after stroke was independently associated with higher age (HR 1.04, *p* = 0.005), elevated WBC (HR 2.72, *p* = 0.002; SHR 2.57, *p* = 0.005), and levels of d-dimers ≥ 3 mg/l (HR 4.40, *p* = 0.008). For the parameters age and levels of d-dimers ≥ 3 mg/l the associations were not significant in competing risk analyses (Table 3).

### Proposal of a score for prediction of risk of diagnosis of cancer within 1 year after stroke

To address the clinical need to identify patients with occult cancer in the context of ischemic stroke, we propose a score model based on the results of the multivariable analysis for prediction of risk of diagnosis of cancer within 1 year after stroke. We included all variables with significant associations in multivariable analysis, i.e. WBC, platelet count, and levels of d-dimers (Table 3) into the score model. The parameter of occurrence of “ischemic lesions in ≥ 2 vascular territories not attributed to cardioembolic stroke” was associated with risk of cancer diagnosis after stroke in univariable but not in multivariable analysis (Table S1, Table 3) which may be due to pathophysiological overlap with elevated levels of d-dimers in the context of hypercoagulability. Still, we decided to include this parameter into the score model.

**Figure 3:**
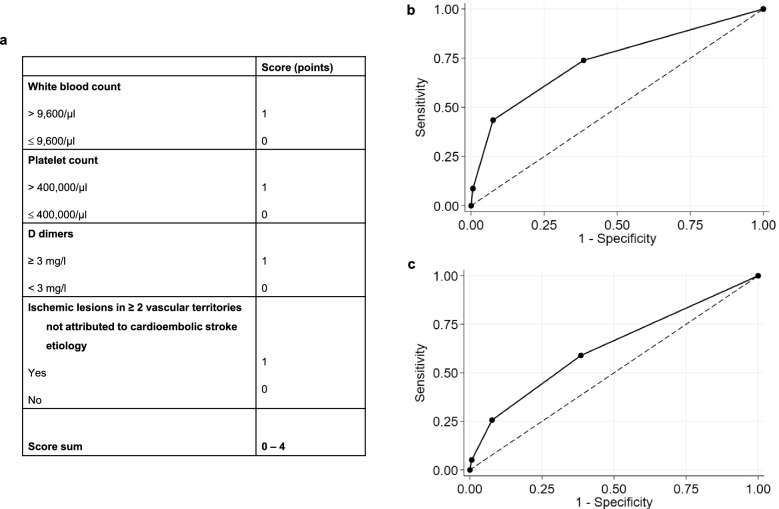
Score model for risk of cancer diagnosis after stroke. (**a**–**c**) Proposed score for estimation of risk of cancer diagnosis after stroke comprising levels of white blood count, platelet count, d-dimers, and occurrence of ischemic lesions in ≥ 2 vascular territories not attributed to cardioembolic stroke etiology (**a**). Receiver operating characteristic curves for the score for prediction of cancer diagnosed within 1 year (**b**) and 3 years after stroke (**c**) with data for all co-variables available for 794 patients, among them 23 and 39 diagnosed with cancer within 1 and 3 years after stroke.

We finally tested a 4-item score of WBC > 9,600/µl, platelet count > 400,000/µl, levels of d-dimers ≥ 3 mg/l and “ischemic lesions in ≥ 2 vascular territories not attributed to cardioembolic stroke” (Fig. 3a) for prediction of cancer diagnosis within 1 year after stroke and 3 years after stroke. The AUC on ROC analysis was 0.73 (95% CI 0.62–0.85) and a score sum of ≥ 2 was associated with a sensitivity of 43% and specificity of 92% for diagnosis of cancer within 1 year after stroke (Fig. 3b). For prediction of risk of cancer diagnosis within 3 years after stroke, this score is less accurate (AUC 0.63, 95% CI 0.54–72) (Fig. 3c).

### External validation of a score suggested by others

Others have suggested a score including levels of d-dimers, hemoglobin and previous or current smoking with an AUC of 0.73 (95% CI 0.65–0.81) for patients younger than 75 years for evaluation of screening for occult cancer in the context of ischemic stroke^6^. We aimed to validate this score in our dataset with complete data available for 407 of 626 patients younger than 75 years, among them 11 and 20 patients diagnosed with cancer within 1 and 3 years after stroke. In our cohort, the AUC for this score was 0.61 (95% CI 0.40–0.82) and 0.57 (95% CI 0.44–0.70) for prediction of cancer diagnosed within 1 and 3 years after stroke, respectively.

## Discussion

Occult cancer in the context of acute ischemic stroke may be underdiagnosed and it remains controversial whether there are relevant risk factors that justify screening for cancer. Here we provide a broad analysis of parameters assessing the risk of cancer diagnosis within 1 and 3 years after acute ischemic stroke without and with consideration of death as competing risk. Elevated levels of WBC, platelets, and d-dimer levels ≥ 3 mg/l were independently associated with cancer diagnosis within 1 year after stroke in multivariable competing risk analysis (Table 3) and occurrence of ischemic lesions in ≥ 2 vascular territories not attributed to cardioembolic stroke were associated with cancer diagnosis within 1 year in univariable analyses. We propose a score model out of these 4 parameters (score sum 0–4) with an AUC of 0.73 (95% CI 0.62–0.85). A score sum of ≥ 2 was associated with a sensitivity of 43% and specificity of 92% for prediction of cancer diagnosis within 1 year after stroke in our cohort for a sum of ≥ 2 (Fig. 3).

A registry study observed a higher risk of stroke in patients with cancer diagnosed up to 1 year prior to stroke, but also with occult cancer diagnosed within 1 year after stroke, for predominantly smoking-related cancer types including lung, colorectal, head and neck, urothelial, pancreatic, kidney, and gastric cancer suggesting smoking as risk factor for both conditions, but also for non-Hodgkin-lymphomas^16^, the types of cancer are comparable with our cohort (Table 1) and with others^5,7,17–19^. Beyond an overlap of risk factors such as age and smoking for both cancer and stroke, there may be also surveillance bias triggered by contact to the health care system due to the work-up of stroke potentially contributing to higher rates of cancers diagnosed after stroke^20^. Still, in some patients, a pathophysiologic relationship between cancer and stroke is suspected with the hypothesis of cancer-mediated hypercoagulability that is reflected by higher rates of venous thromboembolism, elevated levels of d-dimers, and inflammatory biomarkers^21^. Results and methodology of previous studies exploring ischemic stroke and occult cancer were heterogenous and limited by low patient numbers, single-center retrospective design^3^, pre-selection of cohorts, among them one study including only patients with non-disabling stroke^5^, another excluding patients with loss of follow-up, death or incomplete work-up of stroke^7^ while others combined analysis of both patients with known and occult cancer^6,18^ or only included patients that received a cancer diagnosis during work-up of stroke^19^.

The novelty and strength of our methodological approach represents the integration of risk of death as competing risk of cancer diagnosis after stroke respecting age as risk factor for stroke, cancer and death as well as higher post-stroke mortality for patients with preexisting cancer and stroke^5,8,9,22^. It remains uncertain whether intraarterial therapeutic interventions may be beneficial for outcome of patients with cancer and acute ischemic stroke with two studies showing no differences in post-stroke mortality associated with cancer^23,24^, while others suggested higher mortality of cancer patients^22^. Furthermore, we provide a time-dependent analysis of the cohort of patients with cancer diagnosed within 1 and 3 years after stroke. The hypothesis of risk accumulation of cancer diagnosed around the time of stroke was raised by others as well^1,20^ and is confirmed by our observation that a median time of 7.3 months from stroke to cancer diagnosis in the patient cohort diagnosed with cancer within 3 years after stroke (Table 1).

The characteristics of patients associated with risk of cancer diagnosis after stroke as identified by univariable analysis (Table S1), were, at least in part, also suggested by others, among them lower hemoglobin^6,7,25^, higher levels of LDH^18^, and higher age^7,17,26^. Fibrinogen levels were not associated with cancer after stroke in our cohort (Fig. 2, S1) while a study of patients with active cancer diagnosed prior to stroke suggested an association of fibrinogen levels higher than 4.0 g/l^27^, identical with the cut-off used here. Previous studies suggested an association of cryptogenic stroke and ischemic lesions in multiple vascular territories^14,25^ with occult cancer while others did not^7,17^. Here, the occurrence of ischemic lesions in ≥ 2 vascular territories was associated with risk of cancer diagnosis within 1 year after stroke if stroke was not attributed to a concurrent cardioembolic etiology (Table S1. Elevated levels of d-dimers have been related to occult cancer, however with variable cut-offs^6,14,15,25,26^. We confirmed the role of different cut-offs in this context with levels of d-dimers ≥ 3 mg/l^6^ associated with cancer diagnosed after stroke while this was not significant when chosing the ULN (0.5 mg/l) as cut-off (Fig. 2, Table 3). We first show that elevated WBC were independently associated with risk of cancer diagnosis within 1 and 3 years after stroke with and without consideration of death as competing risk (Table 3). WBC were higher in patients with cancer diagnosed within 1 and 3 year after stroke (Fig. 2), also after exclusion of patients with lymphomas and hematologic diseases (Figure S1). Stroke-associated infections as potential confounder for alterations in peripheral blood count were not differentially distributed in patients diagnosed with cancer within 1 and 3 years after stroke (Table 2) and were also not associated with risk of cancer diagnosis within 1 and 3 years after stroke (Table S1). One study found the relative percentage of granulocytes versus total WBC to be associated with preexisting and occult cancer in patients with ischemic stroke, while total WBC and platelet counts were not different^18^. History of venous thromboembolism was associated with risk of cancer within 1 year after stroke but not within 3 years after stroke in univariable analyses (Table S1), and not in multivariable analyses (Table 3). Venous thromboembolism in patients with ischemic stroke was associated with preexisting cancer in previous studies^28–30^ and a prospective trial of patients with idiopathic venous thromboembolism evaluated the role of limited or extended cancer screening activities identifying about 4% of patients diagnosed with cancer within 1 year after stroke^31^.

We suggest a 4-item-score (score sum 0–4) of elevated levels of WBC and platelet count, levels of d-dimers ≥ 3 mg/l and “ischemic lesions in ≥ 2 vascular territories not attributed to cardioembolic stroke” with an AUC of 0.73 (95% CI 0.62–0.85) and a score sum of ≥ 2 was associated with a moderate sensitivity of 43% but high specificity of 92% for prediction of cancer diagnosis within 1 year after stroke in our cohort. (Fig. 3a,b). The less accurate AUC of 0.63 (95% CI 0.54–72) for prediction of cancer diagnosed within 3 years (Fig. 3c) after stroke may be interpreted both by a temporal relationship of cancer and stroke around the time of stroke as discussed before and loss of relevance of measurement of parameters with a longer latency to cancer diagnosis. This is in line with a sensitivity analysis comparing characteristics of patients diagnosed with cancer within 1 year after stroke with those patients diagnosed with cancer more than 1 year after stroke (but within 3 years after stroke) (Table S2) showing higher levels of WBC as well as occurrence of ischemic lesions in multiple vascular territories were associated in patients with earlier cancer diagnosis.

We are aware of the limitation that this score has not been validated in an independent cohort. However, the strength of the score is that all variables are routinely available in most patients with stroke and therefore may be useful in clinical routine. Although the overall incidence of cancer after stroke is low, the suggested score shows a high specifity in our cohort for prediction of cancer after stroke. A score for clinical use intending to screen for occult cancer in the context of ischemic stroke has been suggested by others. This score was derived of a cohort with patients diagnosed with cancer within 12 months before or after the index stroke and build on levels of d-dimers, hemoglobin and previous or current smoking with an AUC of 0.73 (95% CI 0.65–0.81) but was restricted to patients younger than 75 years^6^. We aimed to validate this score in our cohort, but, although limited by low patient numbers, we were not able to confirm accuracy in our patient cohort with an AUC of 0.61 (95% CI 0.40–0.82) and 0.57 (95% CI 0.44–0.70) for prediction of cancer diagnosed within 1 and 3 years after stroke, respectively.

Beyond the limitations that have already been discussed, limitations of our study include the single center and retrospective study design, with heterogeneity of patients and diagnostic procedures, risk of chance findings potentially triggered by multiple analyses and relatively small patient numbers in subgroups. Further limitations represent that there was no standardized protocol for blood sample collection, and that the choice of cut-offs may affect results, notably the use of LLN and ULN as cut-offs as not optimized discriminators for the aim of the study. However, the novelty and strength of our study represents the unbiased and comprehensive analysis of risk factors for diagnosis of cancer after stroke augmented by clinical data and match with a cancer registry database as well as the integration of risk of death as competing risk of diagnosis cancer after stroke.

## Conclusion

We propose to further validate our suggested score of elevated levels of WBC, platelets, levels of d-dimers ≥ 3 mg/l, and the occurrence of ischemic lesions in ≥ 2 vascular territories not attributed to cardioembolic stroke etiology for prediction of cancer diagnosis after stroke and for risk stratification regarding the need of cancer screening activities.

## Supplementary Information

Supplementary Information.

## Data Availability

Anonymized data not published within the article will be shared on reasonable request from any qualified investigator to the corresponding author provided that it is for purposes of replicating results and in line with the requirements of the institutional review board approval.
