# Supplementary material for: A risk model for prediction of diagnosis of cancer after ischemic stroke
Source: Sci Rep. 2023 Jan 3;13:111. doi: 10.1038/s41598-022-26790-y (PMC9810715; doi:10.1038/s41598-022-26790-y)
Supplement: Supplementary file 1 — Supplementary Information. [file 41598_2022_26790_MOESM1_ESM.pdf]

Supplementary material

A risk model for prediction of diagnosis of cancer after ischemic stroke

Katharina Seystahl[1], Dorothee Gramatzki[1], Miriam Wanner[2], Sung Ju Weber[1], Alessia Hug[1], Andreas R. Luft[1, 3], Sabine Rohrmann[2], Susanne Wegener[1], Michael Weller[1]

[1]Department of Neurology, University Hospital and University of Zurich, Zurich, Switzerland

[2]Cancer Registry of the Cantons of Zurich, Zug, Schaffhausen and Schwyz, University Hospital and University of Zurich, Zurich, Switzerland

[3]Cereneo Center for Neurology and Rehabilitation, Vitznau, Switzerland

Table S1: Risk of diagnosis of cancer after stroke without and with consideration of death as competing risk for cancer in univariable analyses

	**Risk of diagnosis of cancer within 1 year after stroke** Data of all 1157 patients if not indicated otherwise				**Risk of diagnosis of cancer within 3 years after stroke** Data of all 1157 patients if not indicated otherwise			
	**Hazard ratio (95% CI)**	**p value**	**Subdistribution hazard ratio (95% CI)**	**p value**	**Hazard ratio (95% CI)**	**p value**	**Subdistribution hazard ratio (95% CI)**	**p value**
**Sex**<br>Male versus female (ref)	1.72 (0.82-3.60)	0.15	1.78 (0.85-3.72)	0.12	1.31 (0.75-2.28)	0.34	1.38 (0.79-2.41)	0.25
**Age**	1.02 (0.99-1.04)	0.13	1.01 (0.99-1.04)	0.21	1.03 (1.01-1.05)	*0.009	1.02 (1.00-1.03)	*0.036
**NIHSS at admission**<br>> 5 versus ≤ 5 (ref)<br>Available data, n (%): 1129 (97.5%)	0.81 (0.41-1.62)	0.56	0.72 (0.36-1.44)	0.35	0.86 (0.50-1.48)	0.59	0.72 (0.42-1.24)	0.24
**Medical history**								
**Venous thromboembolism prior to stroke:**<br>Yes versus no (ref)	3.16 (1.22-8.16)	*0.018	3.14 (1.21-8.13)	*0.018	1.78 (0.71-4.47)	0.22	1.74 (0.68-4.46)	0.25
**Arterial hypertension:**<br>Yes versus no (ref)	0.91 (0.45-1.84)	0.80	0.88 (0.43-1.78)	0.71	1.28 (0.71-2.29)	0.41	1.18 (0.66-2.11)	0.59
**Diabetes mellitus:**<br>Yes versus no (ref)	0.76 (0.27-2.16)	0.61	0.73 (0.26-2.06)	0.55	0.73 (0.31-1.71)	0.47	0.66 (0.28-1.55)	0.34
**Hyperlipidemia:**<br>Yes versus no (ref)	0.76 (0.39-1.50)	0.44	0.78 (0.40-1.54)	0.47	0.75 (0.44-1.28)	0.30	0.77 (0.45-1.32)	0.35
**Active or previous smoking:**<br>Yes versus no (ref)<br>Available data, n (%): 1092 (94.4%)	1.16 (0.58-2.30)	0.67	1.18 (0.59-2.33)	0.64	1.07 (0.63-1.83)	0.80	1.09 (0.74-1.87)	0.74
**Atrial fibrillation**<br>(including diagnoses obtained during work-up of stroke):<br>Yes versus no (ref)	0.49 (0.19-1.27)	0.14	0.44 (0.17-1.13)	0.09	0.44 (0.20-0.97)	*0.042	0.36 (0.17-0.80)	*0.012
**Ischemic stroke:**<br>Yes versus no (ref)	1.35 (0.56-3.25)	0.51	1.33 (0.55-3.19)	0.53	1.43 (0.72-2.84)	0.30	1.34 (0.68-2.66)	0.40

	Risk of diagnosis of cancer within 1 year after stroke<br>Data of all 1157 patients if not indicated otherwise				Risk of diagnosis of cancer within 3 years after stroke<br>Data of all 1157 patients if not indicated otherwise			
	**Hazard ratio (95%-CI)**	**p value**	**Subdistribution hazard ratio (95%-CI)**	**p value**	**Hazard ratio (95%-CI)**	**p value**	**Subdistribution hazard ratio (95%-CI)**	**p value**
**Stroke etiology (TOAST classification)**								
(1) Large artery atherosclerosis versuss all other (ref)	0.49 (0.15-1.60)	0.24	0.51 (0.16-1.67)	0.27	1.09 (0.55-2.17)	0.80	1.16 (0.59-2.29)	0.66
(2) Cardiac embolism versus all other (ref)	0.34 (0.13-0.87)	*0.025	0.32 (0.12-0.82)	*0.018	0.39 (0.19-0.80)	*0.01	0.35 (0.17-0.72)	*0.004
(3) Small vessel disease versus all other (ref)	1.66 (0.69-4.01)	0.26	1.82 (0.76-4.38)	0.18	1.71 (0.86-3.39)	0.13	1.95 (0.99-3.86)	0.06
(4) Other determined etiology versus all other (ref)	n/a	n/a	n/a	n/a	n/a	n/a	n/a	n/a
(5) Unknown etiology or multiple possible etiologies versus all other (ref)	1.34 (0.67-2.69)	0.40	1.31 (0.66-2.62)	0.44	1.17 (0.67-2.04)	0.58	1.15 (0.66-1.99)	0.63
**Ischemic lesions in ≥ 2 vascular territories** Yes versus no (ref)	2.09 (0.97-4.48)	0.06	1.95 (0.91-4.18)	0.08	1.35 (0.68-2.68)	0.39	1.22 (0.61-2.43)	0.57
**Ischemic lesions in ≥ 2 vascular territories without cardioembolic etiology (TOAST 2)**	3.92 (1.83-8.39)	*<0.001	3.69 (1.73-7.88)	*0.001	2.17 (1.06-4.43)	*0.034	2.01 (0.97-4.15)	0.06
**Large vessel occlusion** Yes versus no (ref) Available data, n (%): 1146 (99.0%)	0.74 (0.34-1.59)	0.44	0.65 (0.30-1.40)	0.27	0.70 (0.38-1.29)	0.26	0.59 (0.33-1.10)	0.10
**Stroke-associated infection** Yes versus no (ref)	1.54 (0.67-3.55)	0.31	1.32 (0.58-3.05)	0.51	1.30 (0.63-2.66)	0.47	1.00 (0.49-2.05)	0.99
**Laboratory parameters**								
**D-dimers,** available data, n (%): 794 (68.6%)								
> 0.5 mg/l versus ≤ 0.5 mg/l (ref)	2.43 (0.72-8.19)	0.15	2.23 (0.66-7.53)	0.20	1.80 (0.79-4.07)	0.16	1.55 (0.69-3.50)	0.29
≥ 3 mg/l versus < 3 mg/l (ref)	4.62 (1.90-11.26)	*0.001	3.88 (1.59-9.45)	*0.003	3.47 (1.64-7.32)	*0.001	2.67 (1.25-5.67)	*0.011
**Fibrinogen**, available data, n (%): 944 (81.6%) > 4 g/l versus ≤ 4 g/l (ref)	1.80 (0.85-3.81)	0.12	1.75 (0.83-3.70)	0.14	1.53 (0.86-2.71)	0.15	1.46 (0.82-2.59)	0.20
**White blood cell count** > 9,600/µl versus ≤ 9,600/µl (ref)	4.17 (2.09-8.33)	*<0.001	3.98 (2.00-7.94)	*<0.001	2.48 (1.46-4.21)	*0.001	2.38 (1.40-4.04)	*0.001
**Platelet count** > 400,000/µl versus ≤ 400,000/µl (ref)	7.92 (3.28-19.16)	*<0.001	7.46 (3.09-18.01)	*<0.001	5.68 (2.57-12.58)	*<0.001	5.13 (2.28-11.51)	*<0.001
**Hemoglobin** < 117 g/l (women) and < 134 g/l (men) versus ≥ 117 g/l (women) and ≥ 134 g/l (men) (ref)	2.30 (1.14-4.66)	*0.02	2.08 (1.03-4.20)	*0.041	1.76 (0.97-3.19)	0.06	1.46 (0.80-2.65)	0.21
**C-reactive protein** Available data, n (%): 1152 (99.5%) > 5 mg/dl versus ≤ 5 mg/dl (ref)	1.65 (0.83-3.27)	0.15	1.52 (0.77-3.02)	0.23	1.44 (0.83-2.49)	0.20	1.27 (0.73-2.20)	0.39
**Lactate dehydrogenase** Available data, n (%): 909 (78.6%) > 480 U/l versus ≤ 480 U/l (ref)	3.50 (1.60-7.64)	*0.002	3.14 (1.43-6.90)	*0.004	2.27 (1.17-4.44)	*0.015	1.98 (1.00-3.91)	*0.049

Table S2: Sensitivity analysis comparing characteristics of patients diagnosed with cancer within 1 year after stroke with patients diagnosed with cancer after 1 year and up to 3 years after stroke

	**Patients with cancer diagnosed within 1 year after stroke** Data of 34 patients if not indicated otherwise	**Patients with cancer diagnosed at least 1 year but within 3 years after stroke** Data of 21 patients if not indicated otherwise	**p value**
**Sex: n (%)**			0.31
- Male	24 (70.6%)	12 (57.1%)	
- Female	10 (29.4%)	9 (42.9%)	
**Age** Median (Min-max)	75 (40-95)	72 (55-89)	0.84
**NIHSS (admission)**			0.80
- Available data (n, %)	33 (97.1%)	21 (100%)	
- Median (Min-max)	4 (0-20)	4 (0-20)	
**Medical history: n (%)**			
Venous thromboembolism prior to stroke	5 (14.7%)	0 (0%)	0.07
Arterial hypertension	22 (64.7%)	17 (81.0%)	0.20
Diabetes mellitus	4 (11.8%)	2 (9.5%)	0.80
Hyperlipidemia	15 (44.1%)	9 (42.9%)	0.93
Active or previous smoking	15 (45.5%)	9 (42.9%)	0.85
- Data available	33 (97.1%)	21 (100%)	
Atrial fibrillation (incl. diagnoses obtained during work-up of stroke)	5 (14.7%)	2 (9.5%)	0.58
Ischemic stroke	6 (17.6%)	4 (19.0%)	0.90
**TOAST classification, n (%)**			0.06
(1) Large artery atherosclerosis	3 (8.8%)	7 (33.3%)	*0.02
(2) Cardiac embolism	5 (14.6%)	4 (19.0%)	0.67
(3) Small vessel disease	6 (17.6%)	5 (19.0%)	0.90
(4) Other determined etiology including cancer	7 (20.6%)	0 (0%)	n/a
(5) Unknown etiology or multiple possible etiologies	13 (29.2%)	6 (28.6%)	0.46
**Ischemic lesions in ≥ 2 vascular territories, n (%)**	9 (26.5%)	1 (4.8%)	*0.043
**Large vessel occlusion, n (%)**	9 (27.3%)	5 (23.8%)	0.78
- Data available	33 (97.1%)	21 (100%)	
**Laboratory parameters**			
**D-dimers, n (%)**			
> 0.5 mg/l	20 (87.0%)	12 (75.0%)	0.34
≥ 3 mg/l	7 (30.4%)	2 (12.5%)	0.19
Data available	23 (67.6%)	16 (76.1%)	
**Fibrinogen**			0.50
> 4 g/l, n (%)	12 (42.9%)	7 (33.3%)	
Available data, n (%)	28 (82.4%)	21 (100%)	
**White blood cell count** > 9,600/µl, n (%)	21 (61.8%)	6 (28.6%)	*0.017
**Platelet count** > 400,000/µl, n (%)	6 (17.6%)	1 (4.8%)	0.16
**Hemoglobin** < 117 g/l (women) and < 134 g/l (men), n (%)	12 (35.3%)	3 (14.3%)	0.09
**C-reactive protein** > 5 mg/dl, n (%)	14 (41.2%)	6 (28.6%)	0.35
**Lactate dehydrogenase**			0.08
> 480 U/l, n (%)	10 (37.0%)	2 (12.5%)	
Data available, n (%):	27 (79.4%)	16 (76.2%)	

Figure S1: Laboratory parameters of patients with ischemic stroke without and with cancer diagnosed after stroke excluding patients with lymphomas and hematologic diseases

a-d. Laboratory parameters with the first value available after admission for stroke were analyzed as continuous parameters by Mann-Whitney U test (a, b) and as binary categorical variables using the local standard upper or lower level of normal as cut-offs by Chi-Square test (C, D) comparing patients without and with cancer diagnosed within 1 year (a, c) and 3 years (b, d) after stroke with exclusion of patients with lymphomas and hematologic diseases. Data were shown as scatter dot plots including mean and SD (a, b) and as bars with percentages (c, d) for those patients who had data on the indicated laboratory parameter available, i.e. for 1147 out of 1147 patients for hemoglobin, white blood count and platelets, for 902 patients for lactate dehydrogenase, 1141 for C-reactive protein, for 788 patients for d-dimers, and 935 for fibrinogen.

Figure S1

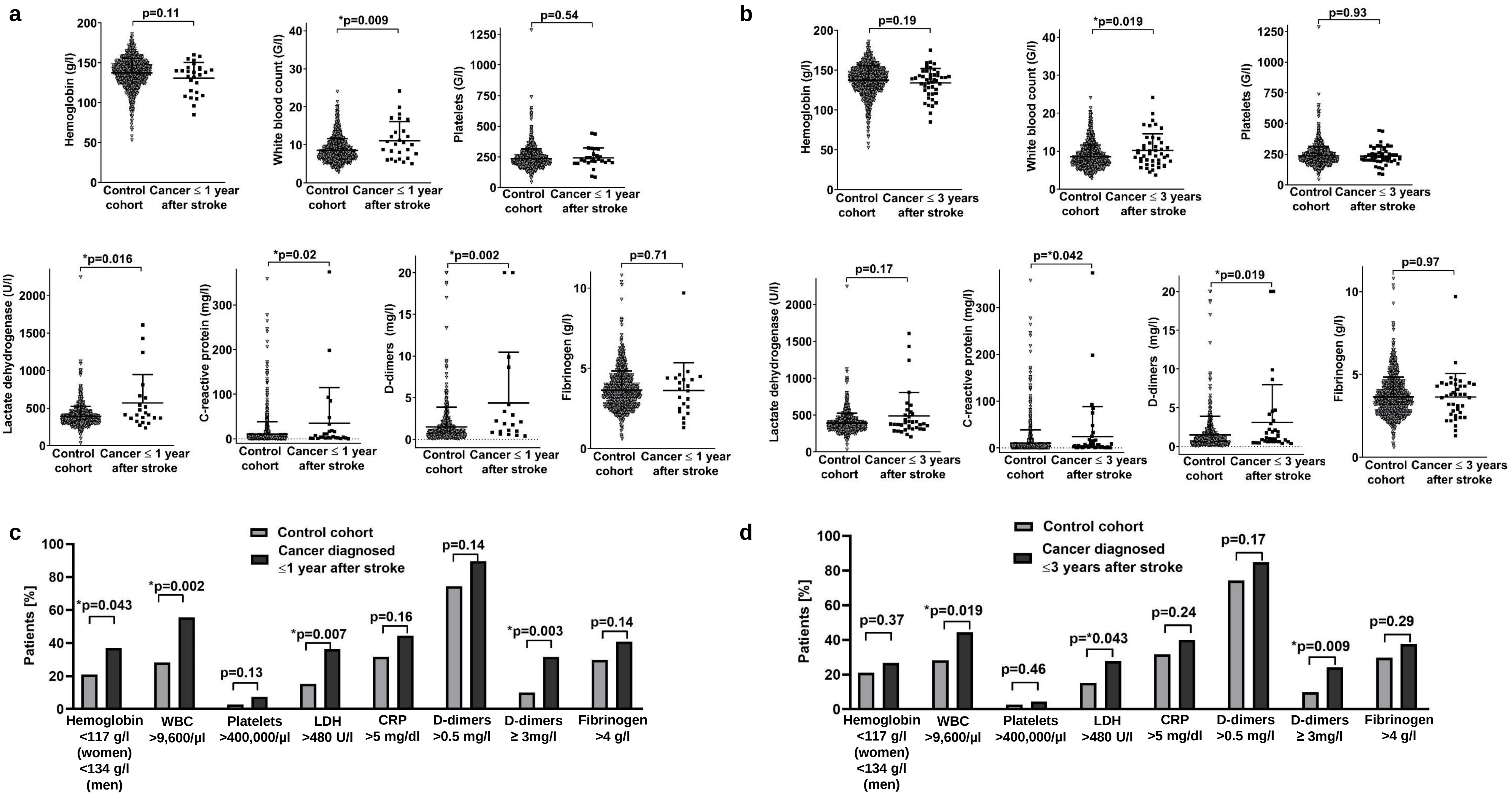

a
p=0.11
Hemoglobin (g/l)
Control cohort
Cancer ≤ 1 year after stroke
*p=0.009
White blood count (G/l)
p=0.54
Platelets (G/l)
*p=0.016
Lactate dehydrogenase (U/l)
*p=0.02
C-reactive protein (mg/l)
*p=0.002
D-dimers (mg/l)
p=0.71
Fibrinogen (g/l)
b
p=0.19
Hemoglobin (g/l)
Cancer ≤ 3 years after stroke
*p=0.019
White blood count (G/l)
p=0.93
Platelets (G/l)
p=0.17
Lactate dehydrogenase (U/l)
p=*0.042
C-reactive protein (mg/l)
*p=0.019
D-dimers (mg/l)
p=0.97
Fibrinogen (g/l)
c
Control cohort
Cancer diagnosed ≤1 year after stroke
Patients [%]
*p=0.043
*p=0.002
p=0.13
*p=0.007
p=0.16
p=0.14
*p=0.003
p=0.14
Hemoglobin <117 g/l (women) <134 g/l (men)
WBC >9,600/µl
Platelets >400,000/µl
LDH >480 U/l
CRP >5 mg/dl
D-dimers >0.5 mg/l
D-dimers ≥ 3mg/l
Fibrinogen >4 g/l
d
Control cohort
Cancer diagnosed ≤3 years after stroke
Patients [%]
p=0.37
*p=0.019
p=0.46
p=*0.043
p=0.24
p=0.17
*p=0.009
p=0.29
Hemoglobin <117 g/l (women) <134 g/l (men)
WBC >9,600/µl
Platelets >400,000/µl
LDH >480 U/l
CRP >5 mg/dl
D-dimers >0.5 mg/l
D-dimers ≥ 3mg/l
Fibrinogen >4 g/l
